# A Practical Perspective on the Roles of Solution NMR Spectroscopy in Drug Discovery

**DOI:** 10.3390/molecules25132974

**Published:** 2020-06-28

**Authors:** Qingxin Li, CongBao Kang

**Affiliations:** 1 Guangdong Provincial Engineering Laboratory of Biomass High Value Utilization, Guangdong Provincial Bioengineering Institute (Guangzhou Sugarcane Industry Research Institute), Guangzhou 510316, China; 2 Experimental Drug Development Centre (EDDC), Agency for Science, Technology and Research (A*STAR), 10 Biopolis Road, Chromos, #05-01, Singapore 138670, Singapore

**Keywords:** NMR, drug discovery, protein structures, protein dynamics, fragment-based drug design, fragment screening

## Abstract

Solution nuclear magnetic resonance (NMR) spectroscopy is a powerful tool to study structures and dynamics of biomolecules under physiological conditions. As there are numerous NMR-derived methods applicable to probe protein–ligand interactions, NMR has been widely utilized in drug discovery, especially in such steps as hit identification and lead optimization. NMR is frequently used to locate ligand-binding sites on a target protein and to determine ligand binding modes. NMR spectroscopy is also a unique tool in fragment-based drug design (FBDD), as it is able to investigate target-ligand interactions with diverse binding affinities. NMR spectroscopy is able to identify fragments that bind weakly to a target, making it valuable for identifying hits targeting undruggable sites. In this review, we summarize the roles of solution NMR spectroscopy in drug discovery. We describe some methods that are used in identifying fragments, understanding the mechanism of action for a ligand, and monitoring the conformational changes of a target induced by ligand binding. A number of studies have proven that ^19^F-NMR is very powerful in screening fragments and detecting protein conformational changes. In-cell NMR will also play important roles in drug discovery by elucidating protein-ligand interactions in living cells.

## 1. Introduction

**Figure 1:**
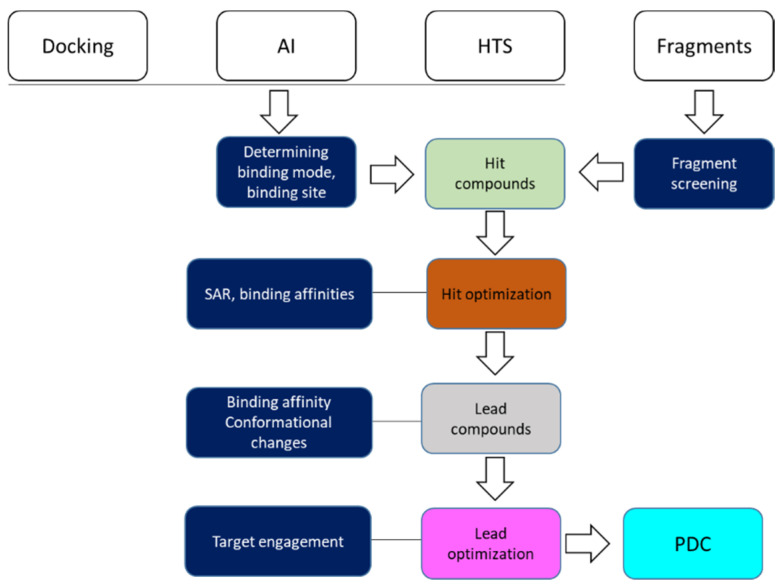
Overview of nuclear magnet resonance (NMR) spectroscopy involved in steps of drug discovery. Hits can come from in silico docking, design with artificial intelligence (AI), high-throughput screening (HTS), and compound fragments. The boxes highlighted in blue are the steps in which NMR can play a role. A preclinical development candidate (PDC) is a compound that is ready for clinical studies.

Nuclear magnet resonance (NMR) spectroscopy is applied to investigate the structures of small molecules in chemistry and large molecules such as proteins from various organisms [1,2,3]. Solution-state NMR spectroscopy is a very attractive tool in drug discovery due to its advantages [4,5]. As samples for analysis are prepared in solution, solution NMR spectroscopy determines the structures of biological molecules under physiological conditions, providing valuable information that helps to understand their function [6]. NMR experiments can be carried out under more rigorous conditions, making it a valuable tool in structural biology [7,8,9,10]. The experiments can be performed under high pressure, at a wide range of temperatures, and in diverse solvents such as organic solvents and a mixture of detergents [11,12,13,14,15,16,17]. To resolve a protein structure using NMR, the following steps are required: sample preparation, data collection, resonance assignment, distance restraint collection, and structure determination [18]. For a protein sample, data acquisition and data analysis are time-consuming, even though data can be obtained in a shorter time with new data-acquisition strategies and automatic data analysis [19,20,21]. Despite such challenges, NMR still plays important roles in structural biology by providing insights into the structures and dynamics of some important biological molecules [22]. NMR is particularly critical for determining the structures of membrane-bound proteins or intrinsically disordered proteins that are not feasible to study using crystallographic approaches [23,24,25,26,27]. As NMR spectroscopy is very sensitive for monitoring protein–protein/ligand interactions, it plays very important roles in such steps of drug discovery as hit identification, hit to lead, and lead optimization (Figure 1) [28]. The hits of a target protein can be obtained by different methods. High-throughput screening (HTS) using biochemical and biophysical assays is frequently applied in this step. Computer-aided docking methods are also very useful for selecting hits from compound libraries. Artificial intelligence (AI) is the simulation of human intelligence using computers to provide rational analysis and prediction for certain processes. AI can play important roles in the process of drug discovery [29]. In the hit identification step, AI is able to provide more reliable hits through machine learning [30]. The hits from different strategies need to be confirmed, and NMR plays critical roles in this step (Figure 1). The theory of NMR experiments and experimental details have been reviewed and described in several publications [1,31,32,33,34,35,36,37,38,39,40,41,42,43,44,45]. In this review, we provide an overview of the application of solution-state NMR spectroscopy in drug discovery. We summarize its advantages in drug discovery, some experimental methods that are able to be utilized in probing protein and ligand interactions, and its future of application in multiple steps of drug discovery.

## 2. Advantages of NMR in Drug Discovery

There are quite a few biophysical methods, such as differential scanning fluorimetry (DSF) [46], isothermal titration calorimetry (ITC) [47], and surface plasmon resonance (SPR) [1,31,32,33,34,35,36,37,38,39,40,41,42,43], that are frequently utilized to elucidate the molecular interactions of macromolecules with ligands [48]. However, NMR spectroscopy is particularly valuable for monitoring the molecular interactions of biological molecules with ligands under physical conditions due to the following advantages. First, a unique feature of NMR spectroscopy is its versatility. Multiple methods can be utilized to monitor protein and ligand interactions (Table 1). Various experiments are powerful for probing protein-ligand binding for NMR, while only one measurement is made for other biophysical methods [5,49]. Both ligand-observed and protein-observed experiments are available for determining protein and ligand interactions [4,5,49,50,51,52,53]. Second, studies are conducted in solutions that are close to physiological conditions and do not require special resins or columns for attaching samples. In addition, the measurements can be taken under such conditions as different pHs, different salt concentrations, and various temperatures [54,55]. Third, NMR method is a label-free technique which can detect interactions of biomolecules with ligands directly without introducing specific chemicals into an assay mixture. It has been noted that chemical modification can also be made in NMR assays. One example is to introduce a fluorine atom to a protein for ^19^F-based NMR experiments [56,57]. Fourth, NMR experiments can be carried out in complex systems [58,59]. Mixtures of ligands can be present in NMR assay tubes. This is very useful in fragment screening, and can save experimental cost and time. Mixtures of proteins can also be studied at the same time, making NMR particular useful for elucidating the effect of a ligand/molecule on protein-protein interactions. Fifth, NMR can be utilized to detect interactions with diverse binding affinities (from mM to nM). NMR is especially powerful for monitoring a ligand’s weak binding to a protein, and dissociation constants can be still obtained for such weak interactions. Although it is not straightforward to determine the binding affinity when a ligand binds to a protein tightly, NMR is still able to determine the ligand binding modes [60,61,62]. Such flexibility makes NMR applicable to different steps of a drug discovery project. Lastly, the recent development of in-cell NMR has made it more valuable in drug discovery. In addition to identifying a hit or confirming an identified hit, in-cell NMR is also useful for confirming the binding mode of a developed compound in living cells, which can be referred to as target engagement [63,64,65,66,67]. It is worth mentioning that NMR is able to measure the binding affinity and determine the ligand binding site from a single experiment, which is a unique feature of NMR spectroscopy. Due to the diversity and flexibility of NMR, careful experimental design is required when a project starts. Suitable experiments should be selected based on the characteristics of a target, such as its molecular weight and dynamic nature.

## 3. Roles of NMR in Drug Discovery

The drug discovery process usually includes such steps as hit identification, hit conformation, hit to lead, and lead optimization. The developed lead will be optimized to a preclinical candidate (PDC) [95] (Figure 1). Solution NMR spectroscopy plays important roles in drug discovery by shedding light on molecular structures, dynamics, and molecular interactions at the atomic level [4,5,45,96]. Therefore, it can be applied in several steps in a target-based drug discovery project (Figure 1). In modern drug discovery projects, the diversity of NMR experiments allows its application in different steps, which is summarized in following sections. Ligand-observed NMR experiments are frequently used in screening and protein-observed experiments play important roles in determining ligand binding modes [53,97]. Protein labeling with isotopes or modification with NMR-active nuclei is required when protein-observed experiments are utilized in drug discovery [12]. It has been noted that a drug discovery project usually has a defined timeline for individual process, which indicates that all NMR-related studies should be conducted in a limited time [98]. Therefore, these NMR studies should be done as early as possible to meet the goals of the project.

### 3.1. Structure Biology

**Figure 2:**
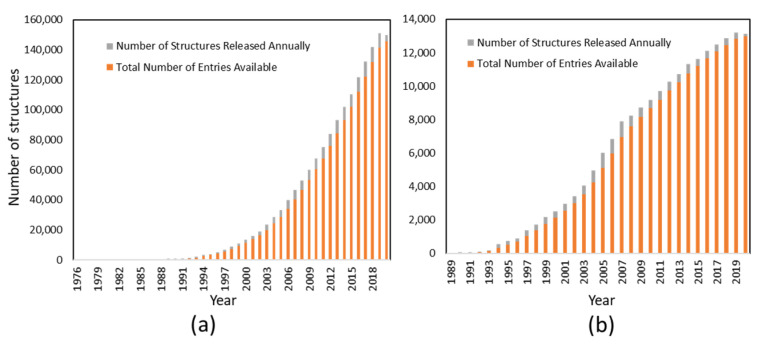
Structures determined by X-ray (**a**) and NMR (**b**) by year. The number of protein structures deposited in the Protein Data Bank (PDB) is plotted against year as of May 2020. The information was obtained from the PDB (https://www.rcsb.org/stats).

Despite its application in determining structures of small-molecular-weight compounds, NMR is still a powerful tool to resolve structures of macromolecules such as proteins and DNA/RNA [22,99]. The newly developed methods make it possible to determine the structures of protein in a short period of time [100]. There are numerous structures resolved by NMR every year, providing valuable insights into structure-based drug design. Although the number of protein structures resolved by NMR is less than that resolved by X-ray crystallography (Figure 2), NMR still plays important roles in the structural biology of many important proteins that are challenging to crystallize [11,12,101]. In addition to determining the structures of water-soluble proteins, NMR spectroscopy has been utilized to determine the structures of membrane proteins and intrinsically disordered proteins which are critical in signal transduction [102,103]. Although the ideal size of a protein for NMR study is below 30 kDa (~300 amino acids), with protein labeling strategies improved, the application of high-field NMR magnets with sensitive probes and diverse restraints makes NMR applicable to resolve the structures of proteins with higher molecular weights [104,105,106,107,108]. The structures obtained by NMR can be used in structure-based drug design and to understand protein-ligand interactions [109]. The time required for structural determination can be reduced by using novel methods such as chemical-shift-guided structural determination using CS-ROSETTA [110,111]. Although NMR plays important roles in structural biology, it is not encouraged to carry out structural studies on a protein when a project has a tight timeline or the target protein is very big, as its backbone resonance assignment might be time-consuming.

### 3.2. Hit Identification and Confirmation

A number of NMR experiments can be conducted to detect protein binding to ligands with diverse affinities (mM to nM). Both ligand-based and protein-based experiments can be utilized to confirm target–ligand interactions (Table 1). Therefore, multiple NMR experiments are able to be carried out in these steps. High-throughput screening (HTS) is a very useful tool with which to identify potent compounds that can be optimized to potent drugs [112]. Biochemical and cell-based assays are frequently applied in screening campaigns. The screened hits usually need to be confirmed with other assays before proceeding to the hit to lead step, as many compounds with measurable inhibitory activity might belong to groups of pan-assay interference compounds (PAINS) [113,114]. NMR spectroscopy, X-ray crystallography, and other physical methods are important for confirming these hits. A titration experiment will be valuable in an NMR-based assay because binding affinity, compound solubility, and specificity can be estimated. All the experiments listed in Table 1 are suitable for confirming hits identified through other methods.

### 3.3. Fragment-Based Drug Discovery

**Figure 3:**
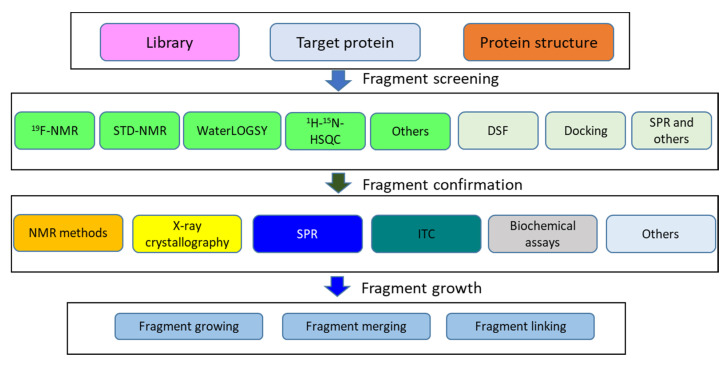
Flowchart of FBDD. A summary of FBDD is given. To start a fragment screening, a compound library and a purified target protein are required. For computational-based screening, the protein structure is needed. NMR plays important roles in fragment screening and hit confirmation. NMR experiments are highlighted in green. “Others” includes experiments such as relaxation-based NMR experiments, SLAPSTIC, etc. (Table 1).

Fragment-based drug design (FBDD) is a strategy used to design potent compounds, and four compounds derived from this way have been approved for clinical applications [115]. A different strategy to HTS, FBDD starts from a fragment that binds weakly to its target. The fragment compound is then grown into more potent compounds [116,117]. A fragment hit can be screened from a library consisting of several hundred to several thousand compounds with diverse structures (Figure 3). Because of the efficiency and low cost of FBDD, this method has been widely used in drug discovery projects [118,119]. Three main steps are involved in FBDD: library selection, screening, and fragment growth [120,121]. Many fragment libraries covering various chemical scaffolds are commercially available [122]. If the size of the fragment library is small, fragment screening can be carried out in a short period of time (within several weeks). The key step in FBDD is to identify suitable hits using sensitive methods. Due to weak binding properties of these fragments, conventional biochemical assays are not applicable for this screening. Therefore, NMR spectroscopy plays a key role in the fragment screening [109]. Ligand-observed NMR experiments such as STD-NMR and WaterLOGSY are frequently applied in these screenings [76,77,123]. ^19^F-NMR experiments have proven to be very powerful in fragment screening, with the following advantages [124]. First, ^19^F-containting fragment libraries are commercially available. Second, ^19^F-NMR has a high signal sensitivity and no background is present in the assay, as biomolecules do not contain fluorine atoms. Third, a mixture of compounds can be used in screening due to the wide range of chemical shifts of ^19^F-containing compounds. Fourth, protein-observed ^19^F-NMR can also be applied to understand protein conformational changes and ligand binding. Lastly, ^19^F-NMR can be readily utilized to rank binding affinities of different compounds, which serves a strategy to screen compounds bound to a specific site when a reference compound is available [83,125]. ^19^F-NMR has recently become the most attractive tool in FBDD because more compound libraries and more sensitive probes have been developed [126,127].

**Figure 4:**
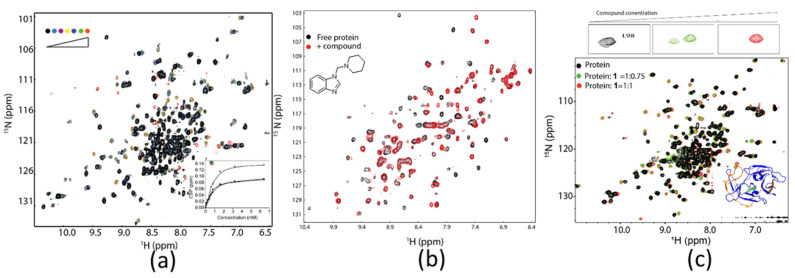
Application of ^1^H-^15^N-HSQC experiments in drug discovery. (**a**) The protease-peptide binding is in fast exchange. The dissociation constant was determined based on titration experiments. The figure is reproduced from Zhang et al. [129] with permission. (**b**) The ^1^H-^15^N-HSQC spectra of TEAD4 in the absence and presence of a compound. The binding is undergoing intermediate exchange. More information can be obtained from Li et al. [130]. (**c**) The ^1^H-^15^N-HSQC spectra of ZIKV protease in the absence and presence of different concentrations of a covalent inhibitor. The binding was undergoing slow exchange, as two peaks corresponding to free and ligand-bound protease were observed when the protein-to-ligand ratio was less than 1. The figure is reproduced from Li et al. [131] with permission.

The heteronuclear experiments such as ^1^H-^15^N/^13^C-HSQC are used frequently in drug discovery, which monitors the chemical shifts of several atoms. Ligand-induced chemical shift perturbation is a sensitive method used to confirm identified hits, locate the position of the ligand binding site on a protein surface, determine dissociation constants for interactions in fast exchanges, and understand structure-activity relationships (Figure 4) [128]. These experiments require a uniformly ^15^N-labeled target, while specifically amino-acid-labeled samples are also applicable. To determine the ligand binding site on the surface of a target, the assignment of cross peaks in a ^1^H-^15^NHSQC spectrum is essential. Backbone resonance usually requires a ^15^N/^13^C or ^15^N/^13^C/^2^H-labeled sample and collection of heteronuclear experiments. With the quantity of resonance assignments deposited in the biological magnetic resonance bank (BMRB) increasing, this type of experiments will play more important roles in drug discovery in the future. Another advantage is that this type of experiments can be utilized to probe protein-ligand interactions in the absence of assignments. Although the binding site cannot be identified without assignments, the protein–ligand interactions are still able to be measured. We have conducted ^1^H-^15^N-HSQC experiments to probe ligand-induced protein conformational changes, determine dissociation constants, map ligand binding sites, and confirm hits in FBDD (Figure 4). Different types of inhibitors may cause different changes in the ^1^H-^15^N-HSQC spectrum. In addition, chemical shift perturbations of residues might be affected by targets, experimental conditions, and binding affinities (Figure 4). For a protein with a known ^1^H-^15^N-HSQC spectrum, this type of experiment is able to play a key effect in FBDD by not only confirming the identified hit, but also screening hits binding to a specific region. A number of potent inhibitors have been developed using this method. One example is the development of inhibitors of KRAS—a validated target that was considered undruggable. Fragments binding to a specific region were identified using ^1^H-^15^N-HSQC experiment and potent inhibitors were obtained based on the identified fragments. In addition, ^1^H-^15^N-HSQC experiments are also a very sensitive method to confirm those identified hits, together with other methods such as thermal shift assay. The limitation of ^1^H-^15^N-HSQC experiments is that the sample has to be isotopically labeled.

### 3.4. Determining Ligand Binding Modes

NMR is also a very useful tool to determine ligand binding modes, even though X-ray crystallography provides high-resolution structures of complexes [132]. To determine the binding mode of a ligand using NMR spectroscopy, some strategies can be adopted in drug discovery, which include understanding SAR based on chemical shift perturbation [81,133], solving solution structures of protein-ligand complexes via intermolecular NOEs, and determining reliable structures of complexes with chemical shift guided docking and limited intermolecular NOEs [134,135,136,137].

#### 3.4.1. Understand SAR in Drug Discovery

Understanding SAR in drug discovery is critical for medicinal chemists to develop more potent compounds. In the absence of co-crystal structures of a target protein with ligands, together with biochemical, biophysical, and docking assays, NMR plays important roles in understanding SAR. One efficient way is to monitor the chemical shift difference of the target induced by different compounds [72]. This method was developed for determining the binding site for a ligand that induced dramatic chemical shift perturbations in the ^1^H-^15^N-HSQC spectrum of a protein [72]. This method was successfully used in the development of peptidic inhibitors of West Nile virus protease [138]. In the study, a potent dipeptide inhibitor was developed, while it is challenging to obtain co-crystal structures of West Nile protease with these inhibitors. We then applied molecular docking and NMR methods to understand the SAR of these inhibitors. Some important groups for enzymatic activity and target binding were identified via this approach. The SAR was later confirmed by resolving the co-crystal structure of a dipeptide inhibitor with Zika virus protease, the structure of which is similar to that of West Nile protease [139]. Quantities of compounds with similar structures were synthesized in two steps of hit to lead and lead optimization. Therefore, NMR fulfils a decisive role in understanding SAR when crystallization of a target is challenging [140]. In addition, NMR spectroscopy provides insight into dynamics of a target in the absence and presence of a ligand, which is not able to be observed in X-ray crystallography. For example, proteases of dengue virus, West Nile virus, and Zika virus contain multiple conformations, and a single conformation can be stabilized via a ligand binding to the protease [75,141]. We have found that fragments behave differently from potent protease inhibitors from NMR studies, as the fragment binding does not affect conformational changes in the protease [142].

#### 3.4.2. Solving Solution Structures of Protein-Ligand Complexes

**Figure 5:**
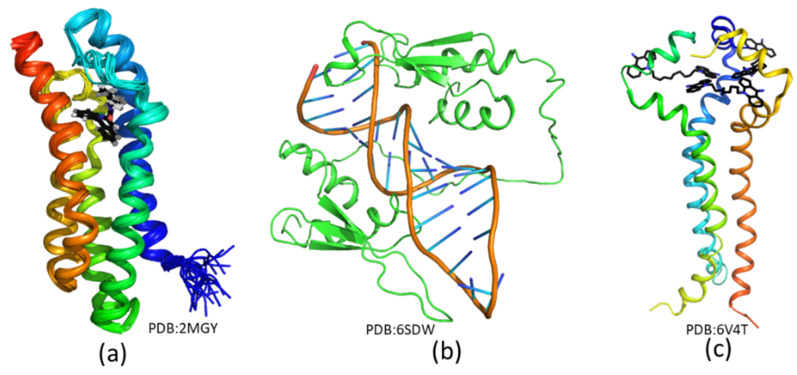
List of several complex structures resolved using solution NMR spectroscopy. (**a**) Solution structure of TPSO in complex with a small-molecule inhibitor [73]. (**b**) Structure of STAU1 dsRBD3/4–sARF1 SBS dsRNA complexes [143]. (**c**) Solution structure of MPER in complex with a small-molecule inhibitor [144]. The small-molecule compounds are shown in black sticks, and the access codes of these structures in the Protein Data Bank (PDB) are indicated in the figure.

The best way to determine the binding modes of ligands is to determine the solution structures of their complexes with target proteins. Various solution structures of protein-ligand complexes have been resolved. Distant restraints such as NOEs between the target protein and ligands are key factors in structure determination [143,144,145]. These restraints can be obtained from NOESY, filtered-NOESY, paramagnetic relaxation, residue dipolar coupling, and other experiments such as cross-linking and biophysical analysis. NMR studies were undertaken of a translocator protein (TSPO), a membrane protein localized on mitochondrial membranes. Solution structures of TSPO in complex with a diagnostic ligand in dodecylphosphocholine (DPC) micelles were obtained using solution NMR spectroscopy (Figure 5a) [73]. Although it is challenging to obtain solution structures of membrane proteins via solution NMR spectroscopy due to the large size of protein–micelle complexes, the success in determining the structure of TSPO proved the importance of NMR in the field of membrane proteins. Structures of proteins in complex with DNA/RNA have also been resolved using solution NMR (Figure 5b). To complete the resonance assignment of the complex, a divide-and-conquer strategy was pursued in resonance assignment [143]. The obtained structure provided useful insights into protein and RNA interactions. Another example is structural studies on molecular interactions between the membrane proximal external region (MPER) of HIV-1 envelope spike and some identified small-molecule fusion inhibitors [144]. The structure revealed that these inhibitors bind to a hydrophobic pocket which is present in the trimeric form of MPER (Figure 5c).

#### 3.4.3. Obtaining Structures through Docking

Due to a defined timeline in drug discovery, it is not ideal to determine solution structures of protein-ligand complexes via conventional methods. Other methods such as bioinformatics or computation-aided structure determination are helpful for obtaining complex structures [146]. Compound-binding-induced chemical shift perturbation in the ^1^H-^15^N-HSQC spectrum of a target protein can be utilized as a restraint to increase accuracy of docking. The availability of high-ambiguity-driven protein-protein DOCKing (HADDOCK) makes it possible to obtain accurate structures of protein-ligand complexes [147]. Orientation of the ligand in the complex can be obtained through the chemical shift difference caused by different compounds with little difference in structures [72]. It is also possible to include NOE restraints in docking. Therefore, intermolecular NOE restraints derived from filtered-NOESY experiments make it possible to determine highly reliable structures via HADDOCK [148]. A study indicated that HADDOCK including NOEs between a protein and ligands was able to guide structure-based drug design of inhibitors [135]. Similar studies were carried out to determine structures of the membrane binding domain of avian sarcoma virus in complex with inositol hexakisphosphate [149]. The residues exhibited chemical shift perturbations and unambiguous NOEs of the target protein and the ligand, which are key factors necessary to achieve reliable models using HADDOCK. We applied this method to characterize interactions between a TEAD protein and fragments. This strategy is very useful for a protein that exhibits detectable cross peaks in its ^1^H-^15^N-HSQC spectrum and for which assignment or partial assignment of the ^1^H-^15^N-HSQC spectrum is required [130]. This method is also useful in drug discovery, especially in FBDD, as a fragment usually leads to modest chemical shift perturbations for a few residues at the binding pocket.

### 3.5. Target Engagement

Target engagement defines the molecular interactions of those compounds developed in drug discovery and their target proteins under physiological conditions [150,151]. As probing ligand interactions with purified proteins may not represent the real interaction physiologically, target engagement is an important step to make sure that the developed compounds are functional as they are designed, and bind to their targets in living cells, corresponding animal models, and patients [152]. To reduce the operational cost, target engagement can be carried out in cell-based assays before moving into animal models. There are a few biophysical and biochemical methods, such as cellular thermal shift assay [153,154] and polarized microscopy [155], that have been adopted in target engagement. Solution NMR spectroscopy might play a role in target engagement by probing protein and ligand interactions in living cells [156].

**Figure 6:**
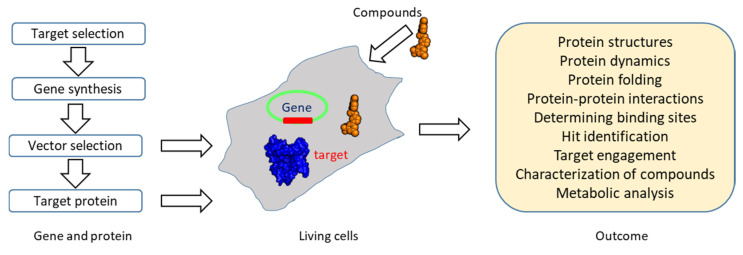
An overview of in-cell NMR. In-cell NMR can be conducted in different cells. Suitable expression vectors (highlighted in green) should be selected for target protein expression. Proteins can also be purified in vitro and transformed/injected into cells using various methods [63,156,169]. The outcome of in-cell NMR is listed in the figure.

Recent studies have shown that structural characterization of proteins can be done in different types of live cells, making it possible to understand the structures, dynamics, and ligand binding of proteins in their native conditions [94]. In-cell NMR spectroscopy is the study of the structure of a protein that is present in living cells without protein purification, wherein an isotopically labeled protein must be overexpressed or delivered into the cells, similarly to the samples used in conventional NMR experiments (Figure 6) [67,103,157,158,159]. In-cell NMR studies such as protein structure determination, protein dynamics, and protein-ligand interactions have been carried out in different cell lines, making the method attractive in structural biology and drug discovery [157,160]. When in-cell NMR was first utilized to determine protein structures in cells, only a few proteins were studied. It can now be applied in different cells such as bacteria, oocytes, insect cells, yeast, and mammalian cells, making it attractive for elucidating protein structures in living cells [161,162,163]. This method is able to probe protein-ligand/protein interactions in living cells, making it a promising tool in drug discovery, as the compounds need to the penetrate cell membrane to interact with their target [164,165,166]. In-cell NMR was shown to be feasible in target engagement by confirming interactions of the antituberculosis imidazopyridine amide (IPA) series with their target in living cells [167]. Ligand-observed experiments such as STD-NMR were conducted in NMR studies. The compounds were confirmed to bind to cytochrome b in living cells, and groups of IPA involving in protein binding were identified, which provided useful information for SAR as well. The mentioned study serves as an example of NMR applied in target engagement; the application of in-cell NMR in structure determination and probing protein-ligand interactions is not described in detail herein. An overview of in-cell NMR is summarized (Figure 6). In addition to in-cell NMR studies, protein-ligand interactions can be monitored in mixtures containing ligand, target, and impurities from cell lysate [168,169]. Although such a study is unable to completely mimic physiological conditions, the effect of impurity on protein-ligand interactions can be evaluated, which is also able to provide some useful information towards understanding target engagement.

### 3.6. Chemical Biology

Chemical biology differs from small-molecule drug discovery, although organic compounds are developed in both fields [170,171]. Chemical biology plays a role in understanding the function of a target protein and serves as an important tool in target validation. Monitoring the conformational changes of a protein induced by a chemical probe and molecular interactions between the protein and the probe are critical in chemical biology [171]. Researchers need to understand both chemical probes and target proteins. Therefore, structures of protein-probe complexes, conformational changes of targets, and metabolic analysis of chemical probes are key elements in chemical biology. Due to some advantages of NMR in probing protein dynamics and ligand binding, it is also critical in chemical biology [172]. In addition to detecting protein-ligand interactions, NMR is a powerful tool to monitor conformational changes of a target in the presence of different types of ligands. G-protein-coupled receptors (GPCRs) have been shown to have diverse conformations when they bind to different types of ligands. ^19^F-based NMR spectroscopy has been successfully applied to probe such changes, which provides direct evidence to support understanding of the conformational changes induced by different ligands [173,174]. As proteins do not contain fluorine atoms, chemical conjugation of ^19^F-labels to the target protein is essential [174], which can be achieved by usage of various chemicals. Linking a fluorine atom to a cysteine residue is most commonly used, and assignments of the ^19^F resonances can be obtained by point mutagenesis. Using one dimensional ^19^F-NMR spectroscopy, ligand-induced conformational changes can be clearly monitored in GPCR [175] and other enzymes [176]. In-cell NMR also plays important roles in chemical biology as it can probe protein–ligand interactions under physiological conditions.

## 4. Perspectives

**Figure 7:**
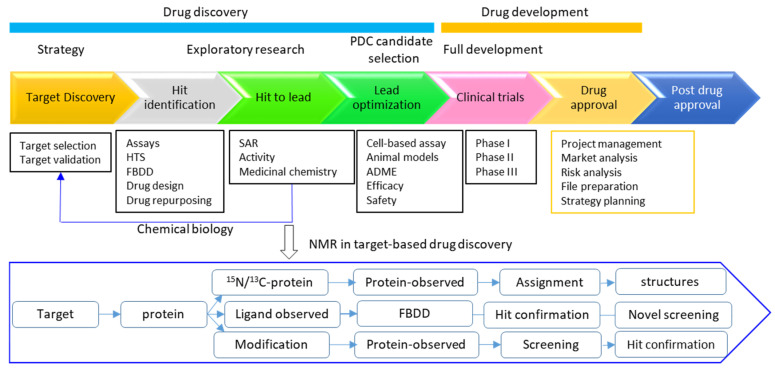
A simplified flowchart for drug discovery and application of NMR in target-based drug discovery. Protein-observed and ligand-observed experiments are applicable for detection of target–ligand interactions, which is dependent on the targets and the screening strategies. ADME means absorption, distribution, metabolism and excretion. SAR indicates structure activity relationship.

To meet the timeline in a drug discovery project, NMR takes a leading role in probing protein-ligand interactions at the early stage of the project (Figure 7). Structure determination of target-ligand complexes can be carried out using HADDOCK guided by chemical shift perturbation and filtered-NOE restraints if it is required. Both ligand-observed and protein-observed NMR strategies can be adopted in screening and hit confirmation, depending on the characteristics of the target (Figure 7).

In addition to hit confirmation, NMR plays important roles in fragment screening as it is the only way to investigate protein-ligand interactions with a wide range of binding affinities (from mM to nM). STD-NMR, WaterLOGSY, relaxation-based NMR, and ^1^H-^15^N-HSQC experiments can be performed in fragment screening (Figure 7). ^19^F-NMR is most attractive in fragment screening, as it is very sensitive and fast in identifying ligands that bind weakly to target proteins.

Competitive screening using NMR provides a novel strategy to identify compounds binding to a known pocket when a reference compound is available. For a target protein with known ligands available, NMR can also be utilized to identify compounds binding to a specific region of the target. ^19^F-NMR is able to identify fragments or large compound mixtures at a high-throughput level.

In-cell NMR becomes very attractive in drug discovery as target-ligand interactions are explored in living cells. Although the timeline allocated for hit identification is generally several months, in-cell NMR is feasible for hit identification for a well-studied target. In-cell NMR is helpful in drug discovery for understanding target engagement of the development of compounds after careful experimental design.

NMR is important in developing inhibitors that affect protein-protein interactions. It is a powerful method to probe protein-ligand interactions when multiple proteins are present in the same assay system. This method is able to allocate the compound binding sites in a mixture containing several proteins. The effect of a compound on the protein-protein complex can be readily monitored in solution.

In summary, solution NMR spectroscopy provides multiple methods to investigate protein structures, conformational changes, protein dynamics, and protein-ligand interactions in solution. It plays important roles in drug discovery. It can also work with other methods to give a thorough view to help in understanding protein-ligand interactions. Suitable protein preparation strategies, sensitive NMR experiments, efficient screening methods, detailed data collection plans, and systematic data analyzing schemes should be designed as early as possible on account of the timeline for a drug discovery project.

## Figures and Tables

**Table 1:** Some NMR experiments used to probe protein-ligand interactions.

Experiments	Applications	References
^1^H-NMR	Screening, protein-ligand interactions	[68,69]
^1^H-NMR, TINS ^1^	Screening	[70]
^1^H-NMR, qNMR	Protein-ligand interactions	[68]
2D HSQC ^1^	Screening, hit confirmation, map ligand binding sites	[71,72]
NOESY ^1^	Hit confirmation, structure determination	[73]
Filtered-NOESY	Hit confirmation, structure determination	[74,75]
STD-NMR ^1^	Screening, hit confirmation	[76]
WaterLOGSY	Screening, hit confirmation	[77,78]
Transferred-NOE	Hit confirmation, structure determination	[79,80]
ILOEs ^1^	Characterizing ligand bindings.	[81]
DOSY ^1^	Hit confirmation	[82]
^19^F-NMR ^1^	Screening, hit confirmation	[56,83,84,85]
FAXS	Screening, hit confirmation	[62,83]
T_2_ CMPG	Screening, hit confirmation	[86]
T_1_ relaxation	Screening, hit confirmation	[86,87]
PRE ^1^	Hit confirmation, structure determination	[88,89]
SLAPSTIC ^1^	Screening	[90]
RDC ^1^	Structure determination	[91]
H/D exchange	Binding characterization	[92]
Cross-saturation	Protein–protein interactions	[93]
In-cell NMR ^1^	Protein structure and ligand binding in living cells.	[94]

^1^ TINS: target immobilized NMR screening; STD: saturation-transfer difference; ILOE: interligand nuclear Overhauser effect; DOSY: diffusion ordered spectroscopy; FABS: ^19^F-NMR includes fluorine chemical shift anisotropy and exchange for screening; CPMG: Carr-Purcell-Meiboom-Gill sequence; PRE: paramagnetic relaxation enhancement; SLAPSTIC: spin labels attached to protein side chain as a tool to identify interacting compounds; RDC: residue dipolar coupling. In-cell NMR may use several NMR methods.
